# Virtual Reality in Marketing: A Framework, Review, and Research Agenda

**DOI:** 10.3389/fpsyg.2019.01530

**Published:** 2019-07-05

**Authors:** Mariano Alcañiz, Enrique Bigné, Jaime Guixeres

**Affiliations:** ^1^Instituto de Investigación e Innovación en Bioingeniería, Universitat Politècnica de València, Valencia, Spain; ^2^Department of Marketing and Market Research, Faculty of Economics, University of Valencia, Valencia, Spain

**Keywords:** virtual reality, marketing, virtual commerce, consumer neuroscience, e-commerce, 3D user interface, presence, psychophysiological assessment

## Abstract

Marketing scholars and practitioners are showing increasing interest in Extended Reality (XR) technologies (XRs), such as virtual reality (VR), augmented reality (AR), and mixed reality (MR), as very promising technological tools for producing satisfactory consumer experiences that mirror those experienced in physical stores. However, most of the studies published to date lack a certain measure of methodological rigor in their characterization of XR technologies and in the assessment techniques used to characterize the consumer experience, which limits the generalization of the results. We argue that it is necessary to define a rigorous methodological framework for the use of XRs in marketing. This article reviews the literature on XRs in marketing, and provides a conceptual framework to organize this disparate body of work.

## Introduction

Digital information and communication technologies (ICT) have, in recent years, significantly improved marketing research, as they have in many other fields, leading to a concept of digital marketing recently defined as “*an adaptive, technology-enabled process by which firms collaborate with customers and partners to jointly create, communicate, deliver, and sustain value for all stakeholders*” (Kannan and Li, 2017). Several studies have analyzed the considerable influence that digital technologies, such as the Internet and social networks, have had on marketing research (Brady et al., 2008; You et al., 2015; Babić Rosario et al., 2016; Kannan and Li, 2017).

One of the most exciting and successful applications of digital marketing is e-commerce, also named e-retail. E-commerce is defined as the process of selling goods and services using electronic media, particularly the Internet (Dennis et al., 2004). In recent years, there has been growing retailer interest in e-retail activities, as the worldwide growth in the e-retail market demonstrates. Its growth is slower in mature markets, such as North America and Western Europe, in comparison to the more rapid growth seen in the developing markets of Asia and Eastern Europe (Nielsen, 2017; Statista, 2017a).

With the advent of more sophisticated technologies that enable high-fidelity reproduction of environments, objects, and persons, e-retailers see Extended Reality (XR) technologies (XRs) as very promising technological tools, able to produce satisfactory consumer experiences resembling those experienced in physical stores. In the present study we use the term XR technologies to encompass virtual reality (VR), augmented reality (AR), and mixed reality (MR). VR is immerses users people into a completely virtual environments, AR provides is creating an overlay of virtual content, but does not allow the user to can’t interact with the (3-D) environment; MR is mixes of VR and the reality, it creating virtual objects that can interact with the actual environment. The use of XRs in retailing to create new computer-mediated indirect experiences has been conceptualized as virtual commerce, or v-commerce (Nguyen et al., 2016).

Extended Reality technologies have already been successfully applied as methodological tools in other scientific disciplines, such as neuroscience (Fox et al., 2009), psychology (Teo et al., 2016), education (Bruer, 2008), medicine (Chicchi Giglioli et al., 2017; McGrath et al., 2018), and human resources (Alcañiz et al., 2018). Therefore, it is not surprising that marketing researchers are showing interest in XRs as a new e-commerce marketing channel with great interactive capacity and totally innovative contents that, to date, have been unavailable to marketing scholars and industry. Prior studies into the use of XRs in marketing are beginning to help us understand the vast potential of these tools for marketing research. The economic impact of VR and AR is forecast to be 29.5 billion United States dollars in 2020. The retail industry’s spending on AR and VR is expected to increase at a compound annual growth rate of 238.7%; it will, thus, become the sector spending most on AR and VR by 2020 (Statista, 2017b).

This new e-commerce channel is of particular interest for the digital native generation. According to recent studies, marketing campaigns using AR have an average dwell time of 75 s (traditional radio and TV ads have dwell times of just 2.5 s), and 71% of shoppers would shop at a retailer more often if they were offered AR (Hackl and Wolfe, 2017).

It is worth emphasizing that there is a growing number of XR related publications and increasing interest in XR among marketing scholars; this will undoubtedly have positive benefits for the field. This article attempts to clarify how XRs are being used in marketing. First, we take a broad perspective of the scientific papers published on the application of XRs in marketing. In recent years, this work has had an important impact on the study of XRs in marketing. However, the inconsistencies within, and the difficulties in interpreting, this growing body of work highlight the need for a systematic approach. From an overall perspective, we found that the field is significantly fragmented in terms of the technologies used and their applications. This tendency can be a motivating factor for the development of a useful framework for classifying the use of XRs in marketing. In the second part of the present study we develop arguments to define the concept of virtual experience in research in marketing (VEM); we then develop and describe a framework for the use of VEMs for research in marketing that highlights the relevant, crucial information that VEM studies must provide.

## Definitions and Framework

### Previous Works on Virtual Experience in Marketing

Through analyzing previous works related to the v-commerce concept (for a recent review, see Bonetti et al., 2018), it has been possible both to understand how VEM has evolved and to revisit the concept in the light of the recent technical advances in XR technologies; this leads us to propose a new definition of VEM.

Some pioneering works about v-commerce used non-immersive graphic interfaces based on computer screens, displaying representations of web-based 2D virtual stores (Gummesson, 1987; Chen and Tan, 2004). Users interacted with the content through traditional input devices (e.g., mouse, keyboard) in a non-natural way. Although these interfaces had low immersion, these works identified several factors that contribute to positive user acceptance of virtual 2D stores, such as product offering, information richness and perceived service quality (Lin and Lu, 2000; Liu and Arnett, 2000).

These 2D web-based virtual stores evolved with the introduction of dynamic 3D product models; these upgraded websites added a new level of buyer–product interaction (Zhang et al., 2004). With this technology, users could interact with the product (e.g., rotate, zoom in/out) using 2D input devices. Several works analyzed the influence of dynamic 3D models on brand attitude, product knowledge and purchase intention (Li et al., 2003; Daugherty et al., 2008).

These two types of experience are limited to an interaction with a virtual replica of the product outside of its traditional sale context, the physical store. Thus, they do not feature other fundamental aspects of user interaction in physical stores, such as navigation, among many others. The experiences also neglected testing or trying on the products, which is important to consumers.

Retailers started to use VR and AR applications at the end of the 1990s. Early pioneers proposed AR technology applications as a research topic (Perid and Steiger, 1998; Brody and Gottsman, 1999; Jones and Biasiotto, 1999), using mobile phones as visual and interaction interfaces with low immersive capabilities. Early VR studies investigated the use of virtual environments in consumers’ homes; they used computer screen visual interfaces and traditional input devices to simulate physical shopping experiences by means of low immersive systems (Gold, 1993; Leinfuss, 1996; Donna and Novak, 1997). In-store VR applications began using screen-based interfaces (Carpenter et al., 1997). The first use of head-mounted display (HMD) interfaces was in 1995, to undertake supermarket redesigns with reduced costs (within Second Life).

Research into VR during the 2000s looked at virtual worlds, which allowed navigation in virtual stores, for example, Second Life© (Linden Labs, San Francisco, CA, United States). When Second Life was launched in 2003, researchers saw it as a useful tool for undertaking social psychology experiments as it offers easy access to large samples. Second Life became a technology of real interest for marketers and advertisers; virtual shopping malls in 3D environments provided interactive and engrossing social interactions with spokes-avatars in a new form of interactive marketing (Kaplan and Haenlein, 2009a). Some works have since analyzed the role of Second Life as a new advertising/communication channel (Barnes, 2011), as a tool for virtual product sales (Jin and Bolebruch, 2009) and for marketing research (Kaplan and Haenlein, 2009b). The virtual experiences offered by online virtual worlds offer low graphical realism, low immersive visual interfaces and unnatural interaction metaphors based on keyboards, a mouse or joysticks. Thus, given the immersive capabilities offered now by XRs, the conclusions drawn from these studies have weak current validity.

Virtual reality has also been used as a tool by test laboratories to obtain metrics to predict consumer behavior in physical stores (Burke, 1996, 2002; Campo et al., 1999; Vrechopoulos et al., 2004, 2009; Breen, 2009; Bigné et al., 2016).

Also during the 2000s, AR applications began to use fishtank interfaces in in-store contexts as virtual try-on tools (Koontz and Gibson, 2002; Barlow et al., 2004; Zhu and Owen, 2008).

In the 2010s, we have witnessed an increase in the number of studies using VR interfaces, but most of them still rely on displays with medium- or low-immersion levels, such as fishtank or large stereo-screen systems.

Pantano and Servidio (2012) used a low immersive stereoscopic powerwall setup (a large screen with stereoscopic vision to investigate consumer reactions to XR technologies. Papagiannidis et al. (2013) used a virtual two-floor fashion clothing store which participants explored through a desktop computer. The participants browsed in the virtual world and undertook assigned tasks using a keyboard or joystick.

Some studies proposed a 3D web-based virtual supermarket to study consumer reactions to marketing strategies, such as price and product labeling (Waterlander et al., 2015), emotional responses to retail environments (Massara et al., 2010) and responses to empty shelf space (Van Herpen et al., 2009). All these studies used low-immersive desktop visual interfaces with mouse-based interactions.

Van Herpen et al. (2016) compared a choice task using VR to a shopping trip in a brick-and-mortar supermarket (with a similar choice task) and a choice task using photographs of products. The virtual supermarket was displayed on a PC and three 42″ LCD screens, which resulted in a 180-degree field-of-view, and participants navigated through the scenario using keyboard and mouse.

A recent study using an immersive VR interface investigated how customers perceived, and if they would purchase, misshapen fruit and vegetables (Verhulst et al., 2017). The participants visualized a virtual supermarket through an immersive HMD and interacted via an Xbox One controller pad. The authors provided a detailed technical description of both the software contents of the virtual environment and the hardware used as visualization and interaction interfaces.

Bigné et al. (2018) compared subjects’ eye gaze patterns during the viewing of a 360-degree video and a 3D display. A more recent exploratory study compared, using a quantitative methodology, the effect of interactivity on emotion during a 360-degree video ad with the effect during a traditional ad (Castellanos et al., 2018). The key question is, does a 360-degree video ad, where the viewer has a free and omnidirectional viewpoint, cause more arousal and positive emotions than the same ad presented in a traditional format, with a fixed point of view?

To our knowledge, these last studies are among the few that use high-immersive visual interfaces based on HMDs for research into marketing, the last being the only one to use a natural motion tracking-based navigation metaphor.

Recently, some studies have started to investigate how consumers react to MR interfaces, such as Microsoft HoloLens (Kalantari and Rauschnabel, 2018).

Table 1 provides a comparative timeline of developments in VEM, indicating the XRs used, the user interfaces, location and disciplinary origins of the research.

**TABLE 1:** Comparative timeline of developments in VEM.

**Chronology**	**XR type**	**User interface**	**VEM location**	**Main findings**
1980–1985	2D web	Non-immersive. Desktop based. Computer screens. Mouse/keyboard	At home	Identified several factors 90 that contribute to positive user acceptance of virtual 2D stores
1985–1990	3D web	Non-immersive. Desktop based. Computer screens. Mouse/keyboard	At home	Positive influence of dynamic 3D models on brand attitude, product knowledge, and purchase intention
Early 1990s	VR and AR	AR: Mobile phone AR interfaces VR: Non-immersive. Desktop based. Computer screens. Mouse/keyboard. First use of HMD for VEM (Stone, 1995)	AR: In-store VR: Mainly at home.	AR: Exploratory uses of augmented commerce. VR: Cost reduction for supermarket redesigns.
2000s	VR (within Second Life) AR (non-mobile phone based)	AR: Screen-based AR interfaces (fishtank) VR: Non-immersive. Desktop based. Computer screens. Mouse/keyboard	AR: In-store VR: At home	Early studies of Second Life as a new advertising/communication channel, virtual product sales and marketing research
Early 2010s	VR	VR: Non-immersive. Desktop based. Computer screens. Mouse/keyboard	At home	Investigated influence of 3D virtual stores on consumer responses (ease of use, enjoyment, store perception, and consumer satisfaction)
2015s	VR AR (cardboard based) MR (first use of MR interfaces in VEM)	First use of immersive VR interfaces. Mainly fishtank interface. Pioneering works proposing HMD interfaces and 3D navigation/interaction devices (laboratory) VR in-store: Cardboard based interfaces AR: Cardboard based interfaces MR: Microsoft Hololens	VR: Laboratory and in-store (cardboard) AR: In-store (cardboard) MR: In-store	Pioneering works using VEM for consumer behavior research. Early studies of QoE using MR

### Definition of Virtual Experience in Marketing (VEM)

In traditional marketing frameworks, consumers learn about products through both direct and indirect experiences. Direct experiences are the physical interactions of the consumer with objects (e.g., products) and subjects (e.g., sellers). This direct communication involves a rich multisensory interaction with products and sellers. Indirect experiences in marketing involve different aspects, such as stores, devices (e.g., computers and smartphones), mass media communication-mediated channels, such as advertising (visual: brochures, billboards, newspapers, and magazines; audio: radio; audio-visual: television) and digital media. One of the most important goals of any e-retailer is to create an optimal shopping experience for the shopper, through computer-mediated communication, predominantly the Internet (Hoffman and Novak, 1996; Dennis et al., 2004). E-commerce has expanded worldwide due to greater internet access, search engines, and different social media formats, such as aggregators, for example, Kayak.com (Dellarocas et al., 2013), online consumer reviews (Zhu and Zhang, 2010) and social networks [e.g., Facebook, Instagram (You et al., 2015)]. Despite this expansion, e-commerce focuses mainly on fashion, travel, books, and music (Nielsen, 2017).

Any consumer experience has its origin in two types of relationship: the buyer–product relationship (Mathwick, 2001) and the buyer–seller relationship (Bagozzi and Verbeke, 2014). Extensive literature identifies what creates a satisfying consumer experience (Szymanski and Hise, 2000). Several advantages/disadvantages of e-retailing for both retailers and buyers have been characterized (Kolesar and Wayne Galbraith, 2000). Interactivity has been identified as a critical advantage of any e-retail system (Merrilees, 2002); it helps the buyer participate, act and learn, and improves feedback from the buyer to the retailer to help him/her produce a very pleasant and enjoyable shopping experience, and develop a close buyer–retailer relationship, thus facilitating good two-way communication.

In contrast, one of the most important disadvantages of e-retailing for consumers is that, up to now, e-retail sites have not been able to reproduce the enjoyable and emotionally important shopping experiences that they enjoy in physical stores. Consumers say that, with e-retail, they do not have as rich an experience as they do in physical stores, which includes multisensory interactions with the product, the store, and salespersons (Lee and Tan, 2003; Bonetti et al., 2018).

The use of XR as a new computer-mediated indirect experience has led to the concept of virtual commerce, or v-commerce (Nguyen et al., 2016). Through XR, the online shopping experience has developed from traditional drag and drop into a cart on 2D websites to a real-time, immersive experience, where users can navigate in virtual shops, and interact with virtual versions of physical products and sellers, just as they do in actual stores.

Some authors define this emerging buyer experience as a virtual experience (Li et al., 2003). Although the term had been schematically used previously (Klein, 1998), Li et al. (2003) characterized a virtual experience as “*a vivid, involving, active, and affective psychological state that consumers encounter when interacting with 3D products in a computer-mediated environment.*” Daugherty et al. (2008), suggested that “*A virtual experience is a simulation of a real or physical experience, which occurs within a computer-mediated environment, and has been constructed to be located between direct (i.e., product trial) and indirect (i.e., traditional advertising) experience along the spectrum of consumer learning.*”

As Hunt (1983a, b) posited, “marketing science is the behavioral science that seeks to explain exchange relationships (p. 12).” Expanding this view, VR can be seen as a technology directed at consummating or facilitating exchanges. More recent approaches, such as service-dominant logic (Vargo and Lusch, 2008), have highlighted the increasing role of consumers in creating value through interaction between products, customer, and sellers. Thus, Allimamy et al. (2019) posited that service-dominant logic and co-creation explain why the use of AR technology reduces customer perceived risks while increasing trust, and importantly, the interaction between buyer and seller are likely to increase.

Virtual experiences and traditional indirect experiences are indirect experiences mediated by a communication channel. Traditional indirect experiences use print communications (e.g., brochures, magazines, and newspapers) and more advanced communication channels, such as TV and 2D websites displayed on computers or smartphones. In traditional print communication, the interaction between consumer and product is entirely static. The perceptual channels rely exclusively on sight; no multiple angle manipulation of the product is possible. The product is presented in a static view, with contextual information embedded in pictures of the product and enhanced by written information containing persuasive messages. This situation is similar to radio ads, where audio is the only sense stimulated. In TV advertising/communication and on 2D websites, the product is presented dynamically with accompanying sensory-rich contextual information. Computers, tablets, and smartphones stimulate almost exclusively the sight and audio senses. The evolution of 2D websites toward 3D multimedia enriched sites enables consumers to interact (e.g., rotate to zoom in/out) in quite similar ways to direct product-buyer experiences. However, the manipulation is indirect, using input devices (e.g., mouse, keyboard) and does not allow the use of more natural interactions (e.g., hand gestures). Moreover, the product is visualized allocentrically (third person). With the addition of stereoscopic interfaces, it is possible to give the viewer a sense of depth; a stereo image of a three-dimensional (3D) scene is displayed on a monitor using a perspective projection coupled to the head position of the observer, known as a fishtank interface (Ware et al., 1993).

The main difference between a virtual experience, and an indirect experience derived from traditional advertising, is that the former provides a richer experience. This difference has its origin in a set of interface characteristics known as affordances. The affordances of human experience in marketing are the interaction expected between consumers and products (Norman, 1998). It is clear that the affordances offered by virtual experiences (virtual affordances) can exceed the affordances the consumer is likely to find in physical environments (physical affordances). Thus, one of the most exciting possibilities of the virtual experience is the fabrication of entirely new situations, impossible to create in the real world, and the development of contexts that will never be experienced by most people in real life. By using XRs we can develop new consumer-product and consumer-context interactions that are not possible in the real world. XRs are not subject to the same space-time restrictions that humans are in the real world. That is, virtual affordances not only match physical affordances, they exceed them. Virtual affordances provide richer communication channels between the consumer and the product than traditional advertising, and much the same interaction with a product as direct experience. In other words, consumers may learn better in a virtual experience than in a direct experience.

**FIGURE 1:**
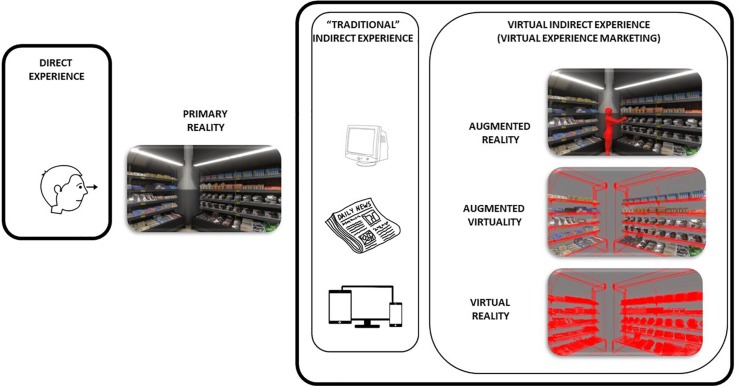
Classification for contents in VEM.

In any virtual indirect experience, the content can be presented to the user in accordance with the “Reality–Virtuality” continuum established by Milgram and Kishino (1994). Inspired by this framework, we propose a new classification for direct and indirect experiences in marketing, shown in Figure 1.

The left side of the continuum depicts the direct observation of a real-world scene – direct consumer experience – through conventional formats (newspaper, radio, TV, computer screen), that is, a traditional indirect consumer experience. The remainder of the continuum shows different situations that occur in virtual experiences. These situations go from AR scenarios, where virtual products are superposed on a real-life scene, to VR scenarios where everything is virtual, passing through augmented virtuality, where the virtual product and the virtual context is augmented with real-life information. We found in the literature several examples of AR experiences, with furniture (Lee, 2017), sunglasses (Grinspan, 2012), make-up (Nesbit, 2014) and fashion clothes (Zugara, 2015). Virtual experiences are also being used in in-store contexts (Tabuchi, 2015; Howland, 2016) in spaces specially designed for immersive experiences (Howland, 2016) and in at-home contexts (Alshaal et al., 2016).

Given this classification, we adopt a more restrictive perspective and define Virtual Experience in Marketing as “any indirect experience in marketing that makes use of XR technologies.” In any VEM, by using XRs, the user is isolated from physical reality by VR aspects or surrounded by virtual elements (AR and MR). Body movements and the sensory flow of the virtual environment are synchronized. Body and head movements are tracked so that the visual and auditory experience reflects the physical body and head movements (Fuchs et al., 2006). XR includes an important new property, the possibility of emulating the eye–hand coordination that occurs in the real-life interaction of the consumer with the product. By using different types of 3D tracking devices, the user can interact with the product as in real life.

In addition, XR allows both allocentric and egocentric (first person) product views. On 3D multimedia-enriched websites, exhibiting products such as cars and real-estate, users can only interact with the products allocentrically. With XR, the product surrounds the consumer, who can have egocentric views, as in real life.

With this proposed definition of VEM, we argue that indirect experiences in marketing mediated by non-immersive technologies (e.g., TV, radio, and 2D websites) cannot be considered VEMs.

We have only just started to explore the benefits to the customer that might be brought by the technical potential of current XRs to generate VEMs.

## A New Methodological Research Framework for Virtual Experiences in Marketing (VEM)

### Research Frameworks in Digital Marketing

Several recent works have proposed different frameworks and taxonomies for research in digital marketing. The framework proposed by Yadav and Pavlou (2014) focuses on marketing in computer-mediated environments. Other frameworks have highlighted other components related to consumer psychology (Lamberton and Stephen, 2016). In methodological issues in marketing analytics by the advent of digital (Wedel and Kannan, 2016), the authors identify XRs as a technological trend that will shape marketing analytics as a discipline as well as marketing analytics education. Indeed, customer experience is recognized as one of the most promising marketing approaches in consumer research (Homburg et al., 2017). This approach complements the digital interactive perspective by emphasizing the customer journey rather than the valuable contribution of the technology itself. Since VR is recognized as an experience in a virtual environment, the role of the experience within this technology must be highlighted. Based on this approach, Farah et al. (2019) discussed how VR could enhance the consumer experience in the consumer journey in retailing. Their findings suggested that VR directly impacts on the users’ sensory elements and therefore enhances the customer experience.

One of the most recent works proposed a framework based on vital touchpoints where digital technologies are having, or are likely to have, a significant impact: environment, company, outcomes, market research, and marketing strategies (Kannan and Li, 2017). Several associated questions for future research are identified at each touchpoint, in which XR evolves as a key enabling technology for the environment touchpoint and, more specifically, for contextual interactions. Kannan and Li (2017) identified XRs as one of the broad categories of technologies that are likely to impact marketing in the near future. In this proposed framework, the authors present several open areas of research for the use of XRs in marketing research, outlining different XR capabilities that can lead to new opportunities. From this analysis, they proposed several open research questions, such as “*With the advent of virtual reality (VR) and augmented reality (AR), contextual interactions become significant. Is the impact of these technologies different in a digital environment vis-à-vis a brick-and-mortar environment? Would they be different for products versus services? How can firms selling customer experiences online (travel, hospitality, vacation packages) benefit from such technology and how can they incorporate the technology in their online decision aid*s? *Can VR and AR technologies increase customer equity?*” (Kannan and Li, 2017).

### A Proposed Research Framework for the Use of VEM in Marketing Research

The literature analyzed in Section “Previous Works on Virtual Experience in Marketing” shows the significant advances in the use of XRs in marketing. We are beginning to realize the enormous potential that XRs have to enhance our understanding of consumer behavior, defining models that analyze the influence that each of the increasingly numerous and complex variables that surround consumers has on their behavior. As previously noted, XR is a tool whose technological capabilities can be of great help to marketing researchers. However, the capability has been adopted and used by only a few pioneering researchers, who are working to understand how XRs can contribute to marketing research. In short, XRs can become commonplace tools in marketing research. Before that, however, it will be necessary to conduct rigorous studies to clarify how XRs might adequately simulate the complex reality that today surrounds the consumer and to analyze the influence that the factors that make up this reality have on his, or her, behavior.

A global analysis of the above-cited works leads us to the following conclusions. First, it is worth emphasizing the growing number of related publications and the increasing interest in XRs among marketing scholars, which will undoubtedly have positive benefits for the field. However, at the global level, the field is significantly fragmented both in terms of the technologies used and in their applications. Also, it is noteworthy that most of the works do not provide enough technical details of the XRs technologies being used. In addition, very few provide an adequate description of the 3D user interfaces used, which is crucial for the reproduction of any XR study.

**FIGURE 2:**
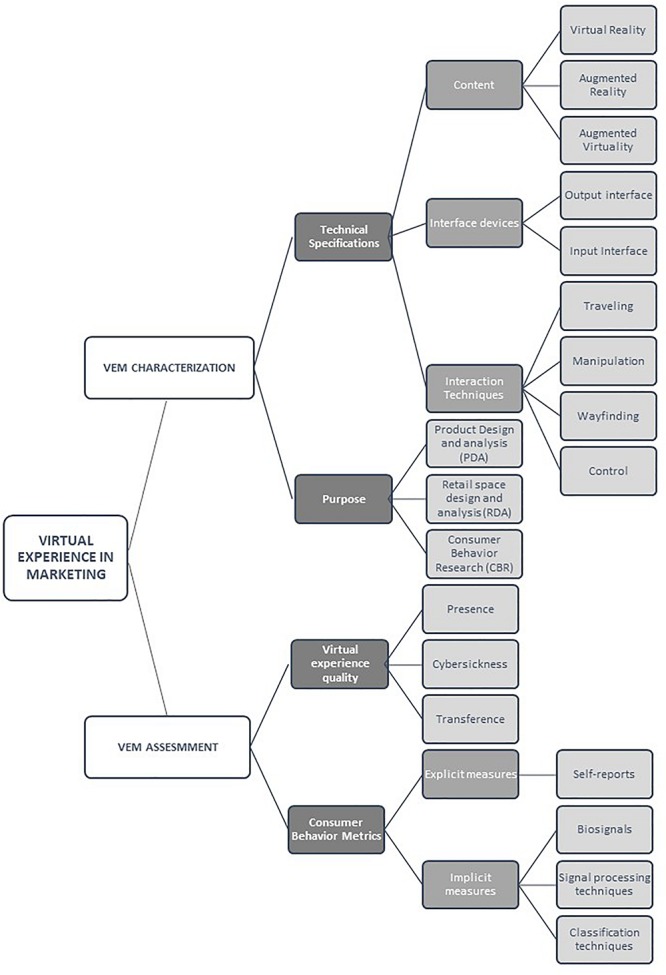
Methodological framework for VEM.

Partial results from previous studies allow us to conclude that XRs can be used to assess several marketing-related constructs. In comparison to retrospective self-reports, XRs have the potential to be a ‘gold standard’ assessment. To reach that stage, however, they must pass robust tests of reliability and validity, which, as yet, is far from the case. For example, applying A/B testing – which is already standard practice in consumer research – to VEM research, would give marketing researchers the tools to investigate the impact of even minor changes in the virtual environments used in VEM experiments. Some recent pioneering studies are starting to consider XR’s potential as an assessment tool. For example, Allimamy et al. (2019) shows that researching, working on, and testing alternative versions of XRs, in this case AR, will likely affect risk perceptions, increase trust, and increase customer willingness to interact with the company that offers AR rather than conventional communication. To achieve this, we argue that it is necessary to define a rigorous methodological framework for the use of XRs for marketing purposes. In this paper, we propose a framework, outlined in Figure 2.

Following a detailed analysis of previous related works, we conclude that the use of XRs in marketing can be classified into three main groups: as a new communication channel for existing or future products (Bonetti et al., 2018), as a tool for testing new store design concepts (Massara et al., 2010), and for studying different aspects of consumer behavior (Verhulst et al., 2017). In all of these groups, XRs are used to observe participants’ responses in laboratory settings, with controlled stimuli, both at behavioral and neurophysiological level, while immersed in virtual environments. XRs can be used for tracking various responses. Using VR low-cost body-motion tracking systems it is possible to measure users’ behavior in real time during virtual experiences. These systems allow the tracking of non-verbal expressions during VR-mediated interactions. In addition, low-cost eye-tracking systems integrated into VR goggles allow the analysis of gaze activity, which provides very valuable information about cognitive states. Miniaturized wearables can be used to obtain psycho-physiological signals which, after processing, provide a valuable indirect source of information related to the brain correlates of participants’ behavior. The synchronization of these signals with the stimuli in the virtual environment (VE) provides the background to cognitively link relevant information in the VE to body responses (Parsons, 2015; Fusaro et al., 2016).

As noted above, VEM facilitates fine-grained recording of implicit human behavior measures, integrated with self-reported descriptions of the experience, to build a more comprehensive and complete model of human responses. Marketing scholars find it difficult, even impossible, to achieve such a high degree of multisensory stimulation, synchronized with human behavior analysis techniques, using other methods. In traditional marketing research, laboratory experimental tasks enable the monitoring of the potentially influential variables that affect subjects’ responses. However, usually, the subject is confronted with controlled stimuli that do not include various variables that are present in real-life situations. Thus, the ecological validity of these methodologies is quite limited. Conversely, it is not easy to study human responses in real-life situations because of the experimenter’s inability to control the stimuli involved in the experience.

The use of VEM for marketing research includes two main processes, the multisensory stimulation of the subject using XRs and the measurement of the subject’s behaviors. For this reason, in our proposed classification we include two main blocks:

**VEM characterization:** That must include the relevant information that characterizes any immersive experience, thus allowing VEM experiments to be replicated and compared.

**VEM assessment:** That must include not only relevant information about the techniques used to analyze subjects’ behaviors but also metrics related to the quality of the user experience.

In the remainder of this section, we provide a detailed analysis of the sub-components included in each main group in our proposed research framework.

#### Technical Specification of VEM

The technical specifications block should contain a detailed description of each component that characterizes any virtual experience (Alcañiz et al., 2003), that is:

• **XR technology:** The XR technology used following the classification outlined in Figure 2, together with a description of the software used and the contents of the virtual environments.
• **Interface devices:** The hardware and software components that present information to the users and allows them to interact with the virtual environment.
• **Interaction techniques component:** The interaction techniques method used to accomplish a given task using the output and input interfaces.

There are many possible choices within each of the three groups of components. Each component has been shown to have a strong influence on the mental processes that give rise to the subjective reality perceived by the user (IJsselsteijn et al., 2001; Clemente et al., 2014; Lorenz et al., 2015; Higuera-Trujillo et al., 2017).

##### Interface device – The output interface

An integral element of any virtual experience is the hardware that presents information to the user. The hardware, known as display interfaces, or output devices, presents information to one or more of the user’s senses through the human perceptual system; the majority focus on stimulating the visual and auditory senses. More recently, several solutions have emerged that stimulate the user’s haptic (i.e., force and touch) senses (Xia, 2018), the olfactory system (Ischer et al., 2014) and taste (Ranasinghe et al., 2012). There are still great technical difficulties in producing portable and high-fidelity output devices for the haptic, olfactory and taste senses. Output devices are shown to have a significant influence on the quality of virtual experiences, in factors such as sense of presence (Baños et al., 2008), immersion (Pausch et al., 1997) and engagement (Buttussi and Chittaro, 2017); and cognitive processes, such as attention, memory, and social relationships (Schnall et al., 2012; Menezes et al., 2017). VEM studies should include a description of the output interfaces used, based on previous works that provide classification taxonomies, such as Krevelen and Poelman (2010) for AR interfaces, Stanney and Hale (2014) for VR visual displays, Bayousuf et al. (2018) for haptic devices, and Stanney and Hale (2014) for olfactory interfaces. For a general classification, see LaViola et al. (2017).

##### Interface device – The input interface

An equally important part of developing a virtual experience is choosing the appropriate set of input devices to allow the user to communicate with the 3D environment, such as 2D desktop input devices (mouse, joysticks), 3D tracking input devices and more natural man–machine interfaces (e.g., voice, natural body movements, bioelectric and brain inputs). Several works provide strong evidence of the effects of input devices on the quality of the user experience (Jerald, 2017) and human performance (MacKenzie and Ware, 1993), among other factors. For a general classification of input interfaces, see LaViola et al. (2017).

##### Interaction techniques

Interaction techniques are software methods that permit the user to interact with the virtual environment by means of interface devices. These techniques can be grouped under selection/manipulation, traveling, wayfinding and system control (LaViola et al., 2017). The interaction techniques used have a profound effect on the quality of the user’s virtual experience, in factors such as presence (Seibert and Shafer, 2018), cognitive load (Varma and Nathan-Roberts, 2017), and human performance (Li, 2017). For a general classification of interaction techniques, see LaViola et al. (2017) and for a more detailed classification of navigation techniques, see Kruijff and Riecke (2018).

It is worth emphasizing that people interacting in the physical world unconsciously handle a series of cues, restrictions, and affordances that are so varied and complex that it is difficult to reproduce them in virtual-reality simulations. Therefore, it is highly recommended that researchers pay attention to the input devices and interaction techniques that generate 3D user interfaces adapted to 3D virtual content. Simply adapting traditional WIMP (Windows, Icons, Menus, Pointer) interfaces to 3D, which is the method followed in the majority of related works, does not provide an adequate solution to the problem. It is necessary to generate 3D user interfaces that not only interact with virtual contents, but also overcome barriers found in the physical world and, what is more important, to analyze the effect that these interfaces have on the consumer experience.

For this reason, it is necessary to carry out experiments that increase our knowledge of the influence that the options in each component group have on aspects of the consumer’s behavior, such as enjoyment, purchase intention, engagement, and consumer learning. To date, very few works address this type of experiment, and those that have done focus almost exclusively on the first group, related to type of content (Tikkanen et al., 2009; Huang et al., 2016; Scholz and Smith, 2016).

On the other hand, as to device components and interaction techniques, the majority of works use low immersive screen-based visualization interfaces coupled with primary input interfaces. Given the rapid evolution of XRs, VEM experiments should use, where possible, the most immersive technologies to emphasize the clear distinction between traditional indirect experiences and virtual indirect experiences. The use of XRs with limited virtual affordances significantly compromises experimental conclusions. In addition, no VEM work addresses the influence of stimulating other sensory channels, such as hearing or smell.

It is beyond the scope of this paper to provide guidelines on how to characterize a virtual experience; for detail on this, see, for example, LaViola et al. (2017). Nonetheless, as a starting point, we argue that any experimental description of a VEM should describe the basic 3D user interface characteristics. It is important in any VEM-related scientific activity to provide detailed information on the options chosen from each of the three groups of components that we propose characterize VEMs. In the VEM related scientific literature we note, in general, a lack of description of the components chosen and, therefore, it is challenging to reproduce the experience to undertake future, enriched versions of the experiments.

#### Purpose of a VEM

A review of the literature on virtual retail reveals that related works can be categorized into three groups, based on the final goals of the studies.

##### Virtual presentations of physical products (PDA type)

The final goal is either to use XR as a new communication channel for existing products (Brody and Gottsman, 1999), or as a means to analyze the consumer’s reactions to mock-ups of future products that do not yet exist (Jaeger and Porcherot, 2017; Rieuf et al., 2017; Van Kerrebroeck et al., 2017).

##### Retail spaces design and analysis (RDA type)

Extended Reality technologies are used to test new store design concepts before construction (Wu et al., 2013; Van Herpen et al., 2016) and to test new product displays and retail layouts (Meißner et al., 2017).

##### Consumer behavior research (CBR type)

A new use of VEM to study in detail the different aspects of consumer behavior. Previous studies proposed VEM as a means of predicting consumer behaviors in real stores using virtual stores (Bressoud, 2013; Van Herpen et al., 2016; Burke, 2018). Other studies analyzed the influence of XR on consumers in aspects such as enjoyment, consumer learning, engagement, and purchase intention (Barnes, 2011; Pantano and Servidio, 2012; Papagiannidis et al., 2013; Torrecilla et al., 2016; Alcañiz et al., 2017; Ausín et al., 2017; Bigné et al., 2018).

#### Virtual Experience Quality Measures

In any experiment in which VEM is used for marketing research, the scientific success of XRs depends on them providing a convincing sense of reality in which participants tend to respond realistically to situations and events portrayed within a virtual replica of a real-life situation and, therefore, give a “response-as-if-real” (RAIR). Therefore, it is highly recommended that RAIR quality experience be assessed. The following measures have proven to be crucial for assessing its effectiveness.

##### Presence measures

Presence is a metric applicable to any XR experience and, thus, to any VEM. It is worth noting that, to date, very few VEM studies have used presence measures. We found in the literature several methods for measuring presence in virtual environments. These are usually classified as either *subjective* or *objective* measures. Subjective measures derive from questionnaires and self-reports solicited during or just after VR exposure. Despite the criticisms aimed at questionnaires, since presence is a qualitative experience, they are the most common approach to its measurement. Among the most used presence questionnaires are the Presence Questionnaire (Witmer and Singer, 1998); the Immersive Tendencies Questionnaire (Witmer and Singer, 1998); and the SUS scale (Usoh et al., 2000). Objective measures of presence are based on correlations of presence with psychophysiological signals, such as heart variability and skin conductance (Meehan et al., 2002; Guger et al., 2004), neuroimaging (Clemente et al., 2013, 2014), behavioral measures and task performance. For a detailed description of presence measurement techniques, see van Baren and IJsselsteijn (2004) and Skarbez et al. (2018).

##### Cybersickness measures

One of the adverse effects suffered by VR users is cybersickness (CS). While several definitions have been proposed, in this work we follow the definition of Stanney: “*CS is a constellation of symptoms of discomfort and malaise produced by VR exposure”* (Stanney and Hale, 2014). Several studies report behavioral indicators of CS, such as the early termination of the VR experience (Kinsella, 2014) and reduced task competence (Nalivaiko et al., 2015). For this reason, we also state that VEM assessments should include a CS measure of the virtual experience. No VEM-based studies, to date, include a CS measure.

As for sense of presence, both objective and subjective measures have been proposed to measure CS. The most commonly used measures of CS are the Simulator Sickness Questionnaire (Kennedy et al., 2003) and the Fast Motion Sickness Scale (Keshavarz and Hecht, 2011). For a more detailed description of assessment methods for presence and CS and their relationships, see Mazloumi Gavgani et al. (2018).

##### Transference

Transference measures are common in VR studies and compare user behavior in a real environment to behavior when interacting with a virtual replica of the environment. When a virtual environment is used as a skills acquisition simulator, as in flight or surgical simulations, the most critical measure of the simulator is its transference capability. The technique is based on a comparison of the user’s two interactions (in the virtual and the real world). To date, transference studies have focused on comparing behavioral measures, such as trajectories, task sequences and task execution time, in fields such as medicine (Latorre et al., 2018) and marketing (Burke, 2018). Very few studies compare cognitive and emotional states by comparing psychophysiological and/or brain activity. This approach would facilitate the development of predictive models of consumer behavior. A recently published study is a first attempt in this regard (Marín-Morales et al., 2018).

#### Consumer Behavior Metrics

Virtual experience in marketing assessments should include measures to evaluate its final goal, that is, to generate consumer behaviors that are as close as possible to reality. For this reason, VEMs must include a set of metrics to evaluate consumer behavior. Although a detailed analysis of all the types of measurement used to characterize consumer behavior is beyond the scope of this paper, we include a list of the metrics most used to date.

##### Explicit measures

Traditionally, in marketing research, the assessment methods most widely used and validated are self-report questionnaires, interviews, and projective measures (Bearden and Netemeyer, 1999). The most used scales can be grouped under the following two categories:

• **Value for the customer:** Customer satisfaction, brand equity, long-term relationships, brand awareness, brand attachment, brand love, customer engagement, and brand engagement.
• **Customer value:** Purchase, retention, brand loyalty, and customer life value (CLV).

##### Implicit measures

To date, most of the theoretical constructs used in consumer behavior are based on explicit measures, such as self-report questionnaires, interviews, and projective measures. The reliability and validity of these techniques can be negatively affected by effects such as social desirability (Grimm, 2010), data interpretation and subject knowledge (Chan, 2009).

A growing number of marketing scholars are paying greater attention to the influence that implicit processes have on consumer behavior (Lee et al., 2007), which has led to the emergence of a new multidisciplinary field, consumer neuroscience (CN). Consumer neuroscience uses neuroscientific insights and methods to enhance the understanding of consumer behavior (Lee et al., 2007; Kenning and Plassmann, 2008; Fisher et al., 2010), using both implicit and explicit measures, thus helping marketing scholars develop more complete and integrated theories of consumer behavior.

In recent times, several techniques for the implicit measurement of consumer behavior have been proposed, based on psychophysiological signals, brain activity measures and/or behavioral measures. For a recent review of the various techniques see, for example, Chark (2018). In Table 2, we summarize the main biometric signals that are being used, the metrics derived from each signal and the psychological constructs related to the metrics.

**TABLE 2:** Most used techniques for implicit measures of consumer behavior.

**Signals**	**What is measured?**	**How is it measured?**	**Which metrics can be derived**	**Related psychological constructs**
ET (eye tracking)	Corneal reflection and pupil dilation	Infrared cameras point toward eyes	Eye movements (gaze, fixation, saccades), blinks, pupil dilation	Visual attention, engagement, drowsiness and fatigue, emotional arousal
GSR (galvanic skin response)	Changes in skin conductance	Electrodes attached to fingers, palms or soles	Skin conductance response (SCR)	Emotional arousal, engagement, congruency of self-reports
FEA (facial expression analysis)	The activity of facial muscles	Camera points toward the face	Position and orientation of the head. Activation of action units (aus). Emotion channels	Emotional valence, engagement, congruency of self-reports
HRV (heart rate variability)	Variability in heart contraction intervals	Electrodes attached to chest or limbs or optical sensor attached to finger, toe or earlobe	Heart rate (hr). Interbeat interval (IBI). Heart rate variability (HRV)	Emotional arousal, stress, physiological activity
EEG				
(electroencephalogram)	Changes in electrical activity of the brain	Electrodes placed on the scalp	Frequency band power, frontal lateralization, event-related potentials, wavelets	Attention, emotional arousal, motivation, cognitive states, mental workload, drowsiness and fatigue
fNIRS (functional near-infrared spectroscopy)	Relative changes in hemoglobin concentration	Electrodes placed on the scalp	Frequency band power, frontal lateralization, event-related potentials, wavelets on prefrontal cortex	Attention, emotional arousal, motivation, cognitive states, mental workload, drowsiness, and fatigue
fMRI (functional magnetic resonance imaging)	Relative changes of cerebral blood flow	Magnetic resonance imaging	Blood-oxygen-level-dependent (BOLD) contrast	Several cognitive and emotional responses
HBT (human behavior tracking)	Body movements (head, hands, rest of the body) and product movements	Cameras placed in front of the subject	Cinematics and dynamics of biomechanical joint movements	Visual attention, engagement, cognitive states, mental workload

The methodology normally used to relate the measurements of biometric signals to consumer behavior metrics is to apply signal processing techniques, followed by computational methods to automatically classify the different consumer behavior metrics. Regarding classification methods, we are witnessing an increased use of machine learning (Mars, 2018) and deep learning techniques (Yu and Deng, 2011; LeCun et al., 2015).

To date, VEM has not been proposed as an experimentation tool to analyze the influence of the different components of products, stores, and sellers on the consumer’s behavior. We suggest that VEM can be used to better understand and model essential elements of consumer behavior, such as purchase intention, engagement, value, and consumer learning. We propose that VEM is a very promising tool to examine various behavioral patterns in dynamic, complex, and realistic situations, that will enhance our knowledge of new models of buyer–product and buyer–seller relationships. In this case, VEMs would not contain virtual replicas of existing products or simulations of future products and stores. On the contrary, they would be used to accurately analyze the influence of general aspects of the product and its contexts on consumer behavior.

## Conclusion

The impact that XRs are going to have on many aspects of our lives is predicted in several studies (Slater and Sanchez-Vives, 2016); almost every aspect related to consumer behavior patterns will be affected by these emerging technologies. Several studies predict that technology-mediated human communications will evolve from today’s smartphones to MR interfaces coupled with artificial intelligence techniques to interpret user activities in most aspects of our lives (Bailenson, 2018) and, more specifically, in our consumer habits and behaviors (Brohm et al., 2017; Grewal et al., 2017). Some recent studies by marketing scholars consider this issue and have proposed a framework for research in digital marketing where VR and AR, that is, XRs, are identified as critical digital technologies that will lead to new marketing opportunities. The capacity of XR to generate new virtual realities will allow the development of controlled laboratory situations in which to study the factors that affect the acceptability of new products and retail spaces and the influence that the different elements that surround consumers have on their decisions.

We predict that the use of interactive and immersive 3D virtual stores will soon become general, and that two purchase channels will coexist. A channel with virtual stores, in which it will be possible to interact virtually with products and virtual sellers, and another channel with physical flagship stores in which it will be possible for the consumer to have a real interaction with real products and real sellers. These physical stores must “compete” with virtual stores and offer the consumer those aspects of the shopping experience that are quite difficult, for now, to provide in virtual stores, such as touching or tasting the product or experiencing the proprioceptive sensations elicited by the product. However, in virtual stores, it will be possible to have at-home consumer experiences, without having to travel, and to collaborate by making purchases, for example, with physically distant friends. These two complementary channels will reinforce the new omni-channel retailing scenario (Neslin et al., 2006; Verhoef et al., 2015). We are in an era of huge advances in XRs. Having been researched for decades, and having been shown to be efficient in many other fields, the ongoing release of consumer-targeted XR hardware platforms signals an opportune moment to develop the next generation of VEMs for widespread dissemination.

Multidisciplinary teams synergizing different scientific disciplines, in our case, engineering, computer science, neuroscience, and marketing, require a period of adaptation so that they can understand their respective needs and capacities. For example, on the one hand, computer science, engineering, and neuroscience researchers, as in this case, have to understand the needs of marketing researchers and their analytical and measurement tools. On the other hand, marketing researchers need to understand the capabilities and limitations of XR technologies.

In this work, we analyze the state of the art of the use of XRs in marketing. As a first result, we conclude that the research field is quite fragmented. Perhaps this is due, in part, to the fact that it is a multidisciplinary field combining several research areas, such as social and technological sciences, with profound methodological differences. Therefore, we argue that it is necessary to define a rigorous and standardized methodological framework. This work makes the first proposal for a framework which allows the classification of research activities in the field.

The vast majority of papers published to date about VEMs have been produced by marketing researchers, who propose the use of XRs to improve our knowledge about consumer behavior. Therefore, it is understandable that these works lack a certain methodological rigor regarding the proposed use of XRs. Published scientific papers that propose the use of XRs, in fields such as education, medicine or training, among many others, include at least a technical description of the interfaces and interaction techniques of XRs. This is essential to ensure the replicability of the experiments and, thus, to make future enhancements to the experiments. These works also include measures to evaluate the quality of the virtual experience, such as presence. This is a fundamental aspect for any work that proposes the use of XRs. As previously noted, in the works published about VEM, there is a lack of detail in the description of both the technical characterization of the proposed XRs and the quality of the virtual experience. Moreover, the lack of clarity in the published works regarding their objectives led us to propose that all VEM studies should be classified based on their key aims. Therefore, we propose to include a detailed description of implicit measures in the proposed framework. Finally, almost all works use metrics based on explicit responses as measures of consumer behavior. Given the growing interest by marketing researchers in implicit measures, it will be necessary to make a distinction between explicit and implicit metrics used in future studies.

The purpose of the proposed methodology is to provide a classification framework that allows the characterization of any VEM study and to provide the minimum information for each of the proposed four groups, that is, technical specifications, purpose, virtual experience quality, and consumer behavior metrics.

## Author Contributions

All authors made substantial contributions to the conception and development of the work. MA is responsible for the general idea of the manuscript. EB and JG participated in drafting the work and revised it in-depth and provided new ideas based on their experience. MA supervised the entire work, reviewed the manuscript, and approved the final version to be submitted.

## Conflict of Interest Statement

The authors declare that the research was conducted in the absence of any commercial or financial relationships that could be construed as a potential conflict of interest.
